# HIV disease is associated with increased biomarkers of endothelial dysfunction despite viral suppression on long–term antiretroviral therapy in Botswana

**DOI:** 10.5830/CVJA-2018-003

**Published:** 2018

**Authors:** Mosepele Mosepele, Mosepele Mosepele, Mohammed Terence, Mupfumi Lucy, Moyo Sikhulile, Lockman Shahin, Bennett Kara, Lockman Shahin, Lockman Shahin, C Hemphill Linda, A Triant Virginia

**Affiliations:** Department of Medicine, Faculty of Medicine, University of Botswana, Gaborone, Botswana; Botswana–Harvard AIDS Institute Partnership, Gaborone, Botswana; Botswana–Harvard AIDS Institute Partnership, Gaborone, Botswana; Botswana–Harvard AIDS Institute Partnership, Gaborone, Botswana; Botswana–Harvard AIDS Institute Partnership, Gaborone, Botswana; Botswana–Harvard AIDS Institute Partnership, Gaborone, Botswana; Bennett Statistical Consulting Inc, Ballston Lake, New York, USA; Division of Infectious Diseases, Brigham and Women’s Hospital, Boston, MA, USA; Department of Immunology and Infectious Diseases, Harvard TH Chan School of Public Health, Boston, MA, USA; Division of Cardiology, Massachusetts General Hospital and Harvard Medical School, Boston, MA, USA; Division of General Internal Medicine and Division of Infectious Diseases, Massachusetts General Hospital and Harvard Medical School, Boston, MA, USA

**Keywords:** human immune deficiency virus, endothelial dysfunction, inflammation, monocyte activation, atherosclerosis, Africa, cardiovascular disease

## Abstract

**Background:**

Untreated HIV infection is associated with increased biomarkers of endothelial dysfunction. However, the predictors and degree of endothelial dysfunction among virally suppressed HIV–infected adults on long–term antiretroviral therapy (ART) have not been well studied in sub– Saharan Africa (SSA).

**Methods:**

We enrolled 112 HIV–infected adults with virological suppression on long–term ART and 84 HIV–uninfected controls in Botswana. We measured plasma levels of markers of endothelial injury [soluble vascular adhesion molecule 1 (VCAM–1), intercellular adhesion molecule 1 (ICAM–1) and E–selectin] and plasma levels of biomarkers of inflammation [interleukin 6 (IL–6)] and monocyte activation (sCD163). Baseline traditional cardiovascular disease (CVD) risk factors and bilateral common carotid intima–media thickness (cIMT) were also available for all participants. We assessed whether HIV status (despite virological suppression on ART) was associated with biomarkers of endothelial dysfunction after controlling for traditional CVD risk factors in linear regression models. We additionally assessed the association between IL–6, sCD163 and cIMT with endothelial dysfunction in separate multivariate linear regression models, controlling for cIMT, among virally suppressed HIV–infected participants only.

**Results:**

In multivariate analysis, HIV infection was significantly associated with increased VCAM–1 (p < 0.01) and ICAM–1 (p = 0.03) but not E–selectin (p = 0.74) levels. Within the HIV–positive group, higher sCD163 levels were associated with decreased ICAM–1 and E–selectin (p < 0.01 and p = 0.01, respectively) but not VCAM–1 (p = 0.13) levels. IL–6 was not associated with any of the biomarkers of endothelial dysfunction.

**Conclusion:**

HIV disease was associated with biomarkers of endothelial dysfunction among virally suppressed adults in Botswana on long–term ART after controlling for traditional CVD risk factors. Future work should explore the clinical impact of persistent endothelial dysfunction following longterm HIV viral suppression on the risk of CVD clinical endpoints among HIV–infected patients in this setting.

Biomarkers of endothelial dysfunction are elevated among untreated HIV–infected patients in Africa,^1^–^4^ and have been noted to be persistently elevated among non–African HIV–infected patients after initiating antiretroviral therapy (ART).^5^–^8^ However, little is known about endothelial dysfunction among virally suppressed HIV–infected patients following long–term ART versus controls in the African setting. Similarly, the independent effects of chronic inflammation and monocyte activation on endothelial dysfunction have not been widely assessed among African patients following sustained viral suppression.

Increased expression of endothelial adhesion molecules is a marker of endothelial dysfunction. Commonly studied endothelial adhesion molecules that predict atherosclerotic disease in the general population include intercellular adhesion molecule 1 (ICAM–1), vascular cell adhesion molecule 1 (VCAM–1) and selectins (P– or E–selectin).^9^–^11^ These molecules work in concert to promote development of atherosclerotic disease, with selectins facilitating activated leukocyte rolling and the adhesion molecules permitting adhesion and passage of leukocytes into the sub–endothelial space (site for atheroma formation).^12^ Expression of these molecules among virally suppressed HIV–infected patients has been linked to both systemic and arterial inflammation as well as immune activation.

HIV–infected patients experience excess arterial inflammation,^13^–^16^ which has been linked to an increase in cardiovascular events.^17^ One of the main drivers of arterial inflammation is activated monocyte production of interleukin 6 (IL–6).^18^,^19^ IL–6 has been strongly associated with atherosclerotic disease^20^–^22^ and all–cause mortality among HIV–infected persons in Botswana and elsewhere.^22^,^23^ Similarly, soluble CD163, a haemoglobin scavenger protein released from activated monocytes, has been linked to arterial inflammation and all–cause mortality among HIV–infected persons.^24^,^25^ More recently, activated non–classical monocytes and VCAM–1 were significantly associated with degree of carotid intima thickness in a cohort of virally suppressed patients in the United States of America.^26^ Exactly how these markers of inflammation and monocyte activation impact on endothelial function in sub–Saharan Africa (SSA) is not widely known.

We therefore sought to compare the degree of endothelial dysfunction among virally suppressed HIV–infected participants on ART in Botswana with that of HIV–uninfected controls, who had similar age and gender distributions, after controlling for traditional cardiovascular risk factors. Within the same setting, we sought to further assess whether biomarkers of inflammation (IL–6) and monocyte activation (sCD163) or carotid intima–media thickness (cIMT) were associated with biomarkers of endothelial dysfunction among virally suppressed HIV–infected participants. We hypothesised that HIV–infected participants have elevated biomarkers of endothelial dysfunction when compared with an HIV–uninfected control group. Additionally, we hypothesised that both IL–6 and sCD163 independently drive excess endothelial dysfunction among HIV–infected participants.

## Methods

Participants were randomly selected from a larger cross-sectional study whose main aim was to assess sub–clinical carotid atherosclerosis and immune activation among adult HIV–infected patients compared to HIV–uninfected controls in Gaborone, Botswana. All participants self–identified as black Africans and were enrolled between February 2014 and April 2015. HIV–infected participants between 30 and 50 years of age (roughly balanced by gender) were recruited from Princess Marina Hospital Infectious Disease Care Clinic (PMH–IDCC).

All HIV–infected patients were required to have documented dual–positive HIV enzyme–linked immunosorbent assay (ELISA) testing or pre–treatment HIV RNA > 400 copies/ml, a minimum six months of documented use of ART, no change in antiretroviral regimen in the six weeks preceding enrolment, and documented HIV RNA < 400 copies/ml throughout the six months prior to enrolment. ART in all participants consisted of two nucleoside/tide reverse transcriptase inhibitors (NRTI) plus either a non–nucleoside reverse transcriptase inhibitor (NNRTI) or a ritonavir–boosted protease inhibitor (PI). Patients on national salvage regimen consisting of two NRTIs plus combination of a ritonavir–boosted PI and an integrase inhibitor were eligible to participate. We also recorded the lowest CD4 count prior to ART initiation (nadir CD4) and the last recorded CD4 count prior to ART initiation (baseline CD4 count). All HIV RNA results were available from laboratory testing during routine clinic visits. HIV–uninfected participants were enrolled at the main Gaborone voluntary HIV testing centre and were required to have documented same–day dual–negative HIV ELISA testing.

At enrolment, all participants provided written, informed consent to participate. Each participant had all study–related procedures completed on the day of enrolment. A targeted interview and review of medical records were performed to obtain CVD risk history, including prior CVD events, family history of CVD, diabetes mellitus or use of antidiabetic medications, hypertension or use of antihypertensive agents, statin use, cigarette smoking, and chronic kidney disease. Medical records were also reviewed to obtain complete HIV disease history, including diagnoses, pre–antiretroviral therapy and enrolment HIV–associated laboratory results, and complete anti–retroviral treatment history.

Furthermore, a focused physical examination was done to obtain resting bilateral arm blood pressures and calculate body mass index (BMI). Non–fasting plasma samples previously obtained and frozen on the day of enrolment were used to measure levels of biomarkers of endothelial dysfunction, inflammation and monocyte activation. Non–fasting lipid profiles and glycosylated haemoglobin levels were available from prior analysis of samples drawn on the same day as the enrolment, when all study–related procedures were completed (including storage of plasma that was thawed for the current analysis).

The study protocol was approved by the Botswana Ministry of Health Human Research Ethics Committee, Princess Marina Hospital Ethics Committee and Partners Human Research Committee in Boston, MA, USA.

The degree of endothelial injury was assessed using ELISA commercially available kits from R&D systems© products supplied by Bio–technec, Abingdon, United Kingdom. The ELISA kit manufacturer’s instructions were followed to ascertain levels of the following biomarkers: human soluble E–selectin (CD62E) quantikine ELISA [lower limit of detection (LOD) 0.1 ng/ml, upper LOD 8 ng/ml], human soluble vascular cell adhesion molecule 1 (sVCAM–1)/(CD106) quantikine ELISA (lower LOD 6.3 ng/ml, upper LOD 200 ng/ml) and human intercellular adhesion molecule 1 (sICAM–1)/(CD54) non–specific allele quantikine ELISA (lower LOD 0.6 ng/ml, upper LOD 40 ng/ ml) kits.

Soluble CD163 and IL–6 levels were measured using ELISA (Trillium Diagnostics, Bangor, ME, USA & R&D systemsc products) according to the manufacturer’s instructions. All participants had VCAM–1 and ICAM–1 results above the LOD. All participants had E–selectin levels above the LOD, however, E–selectin was available in only 90% of the HIV–infected participants and 58% of the HIV–uninfected controls due to limited access to testing reagents. All testing was done on thawed EDTA plasma samples at the Botswana–Harvard HIV Reference Laboratory, Gaborone, Botswana.

We used mean common cIMT as a summary measure of observed degree of atherosclerosis among HIV–infected participants. Briefly, mean cIMT was ascertained at the distal 1cm of the common carotid arteries on images obtained in B–mode along the lateral, anterior and posterior longitudinal sections of the common carotid artery bilaterally (right and left common carotid) as per the 2008 American Society of Echocardiography Carotid Intima–Media Thickness Task Force protocol.^27^ A Sonosite M–turboc ultrasound machine (FUJIFILM Sonosite Inc, Bothell, WA, USA) connected to an 8–12–MHz linear probe was used to obtain still images at the beginning of the R wave, and sonocalc (version 5 of 2011) was used to measure cIMT in auto–mode as per the manufacturer’s instructions.

## Statistical analysis

Baseline characteristics were compared using the two–samples t–test for continuous variables and Fisher’s exact test for categorical variables. All biomarkers and cIMT were initially compared between HIV–infected versus HIV–uninfected participants. The association between HIV status and level of each biomarker of endothelial dysfunction was assessed in univariate and multivariate linear regression, with the following variables selected a priori in the multivariate model: age, gender, BMI, mean arterial pressure (MAP), total cholesterol and glycosylated haemoglobin levels, smoking status (current non–smoker vs smoker) and statin use. Both the biomarkers of endothelial dysfunction and sCD163 and IL–6 levels were assessed for deviations from the normal distribution and if noted to be skewed, data were log transformed.

During initial model building to assess the association between HIV status and endothelial dysfunction, observed associations were assessed both visually in graphical plot and formally (the latter by including a quadratic term in the model and testing for significance). These additional assessments are not reported for ICAM–1 and VCAM–1 because the overall level of significance of the association was unchanged, and similarly it was not reported for E–selectin due to high rates of missing data.

Finally, among HIV–infected participants only, IL–6 and sCD163 were assessed for associations with endothelial dysfunction in univariate models, and then in multivariate models adjusted for each other (IL–6 vs sCD163) and cIMT (as a composite surrogate marker of observed burden of atherosclerosis). If any outliers were noted during preliminary data review (which occurred for IL–6 and log sCD163), sensitivity analyses were performed excluding these observations, and findings were reported if they were different. Furthermore, the three covariates (IL–6, sCD163 and cIMT) were assessed for collinearity and any detected high correlation ( > 0.3) was considered to not impact on the analyses if model estimates remained similar between univariate and multivariate models (i.e. estimates were in the same direction and with similar significance level).

As in the initial model building above, the association between predictors (IL–6, sCD163 and cIMT) for individual models (ICAM–1, VCAM–1, E–selectin) was assessed both visually in graphical plot and formally (the latter by including a quadratic term in the model and testing for significance). A p–value < 0.05 was considered significant in all analyses. All analysis was done in SASc, version 9.4 (SAS Institute, Cary, North Carolina, USA).

## Results

Among the participants, 112 were HIV infected with HIV–1 RNA < 400 copies/ml (51% were female, mean age 40 ± 5 years) and 84 were HIV–uninfected controls (45% female, mean age 38 ± 5 years). HIV–infected participants had a mean HIV disease duration of 9.8 ± 3.1 years and had been on ART for 8.5 ± 2.7 years, with the majority (73%) still on first–line NNRTI–based ART as per the Botswana national ART guidelines. In addition to an NNRTI (73%) or protease inhibitor (PI) (26%), each participant ART regimen also contained Tenofovir/Lamivudine (49%), Zidovudine/Lamivudine (47%), Abacavir/Lamivudine (2%) or Lamivudine (2%) (data not shown in table). Table 1 provides baseline characteristics including CVD risk factors.

**Fig. 1:**
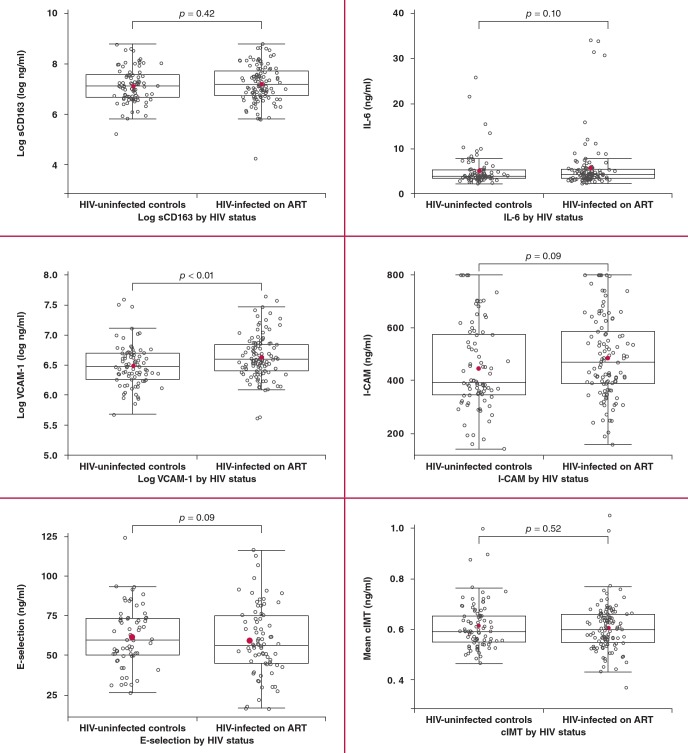
Comparison of biomarkers and cIMT between the study groups. The horizontal bars of the whiskers represent the 10th and 90th percentile, while the upper and lower boundaries of the boxes represent the first and third quartiles. All comparisons are between HIV–infected patients on ART and HIV–uninfected controls. From left to right for each pair of box plots, the top depicts sCD163 (ng/ml) and IL–6 (ng/ml), the middle depicts log VCAM–1 (ng/ml) and ICAM–1 (ng/ml), and the bottom depicts E–selectin (ng/ml) and cIMT (mm). VCAM–1, vascular cell adhesion molecule; ICAM–1, intercellular adhesion molecule; cIMT, carotid intima–media thickness.

Among the biomarkers of endothelial dysfunction in unadjusted analysis, only VCAM–1 was elevated among HIV–infected participants when compared to HIV–uninfected participants (p < 0.01), while ICAM–1 and E–selectin did not differ by HIV status, as shown in Fig. 1. Similarly, sCD163, IL6 and cIMT did not differ by HIV status. The reported p–value for IL–6 includes five HIV–infected outliers; exclusion of these did not alter the direction of the association.

**Table 1:** Demographics and clinical characteristics of HIV-infected participants and HIV-uninfected controls

*Variables*	*HIV-infected, n = 112^a^*	*HIV-uninfected, n = 84^a^*	*p-value*
Age (years)	40 ± 5	38 ± 5	**0.02**
Female, n (%)	57 (51)	38 (45)	0.47
Cigarette smoking, n (%)			
Never	70 (63)	56 (67)	< **0.01**
Prior smoker	36 (32)	12 (14)	
Current smoker	6 (5)	16 (19)	
Family history			
Myocardial infarction, n (%)	1 (1)	4 (5)	0.17
Stroke, n (%)	8 (7)	18 (21)	**0.01**
Personal history			
Diabetes mellitus, n (%)	1 (1)	0 (0)	1.00
Hypertension, n (%)	15 (13)	10 (12)	0.83
Chronic kidney disease, n (%)	4 (3)	0 (0)	0.14
Dyslipidaemia, n (%)	9 (8)	2 (2)	0.12
Medications (current)			
Anti-hypertensives, n (%)	15 (13)	9 (11)	0.66
Statins, n (%)	6 (5)	1 (1)	0.24
Anthropometric data			
Systolic blood pressure (mmHg)	129.7 ± 15.1	130.4 ± 17.0	0.75
Diastolic blood pressure (mmHg)	84.6 ± 11.5	84.6 ± 13.3	0.97
Body mass index (kg/m^2^)			
Underweight (< 18.5), n (%)	10 (9)	8 (10)	0.98
Normal weight (18.5–< 25), n (%)	58 (52)	43 (51)	
Overweight (25–< 30), n (%)	28 (25)	23 (27)	
Obese (≥ 30), n (%)	15 (14)	10 (12)	
CVD risk laboratory tests			
Total cholesterol (mmol/l)	4.8 ± 1.2	4.5 ± 1.2	0.06
LDL cholesterol (mmol/l)	2.9 ± 1.0	2.6 ± 1.0	**0.02**
HDL cholesterol (mmol/l)	1.4 ± 0.5	1.4 ± 0.4	0.87
Triglycerides (mmol/l)	1.4 ± 0.8	1.2 ± 1.5	0.41
Glycosylated haemoglobin (g/dl)	5.3 ± 0.4	5.5 ± 1.0	**0.01**
HIV parameters			
HIV disease duration (years)	9.8 ± 3.1		
Duration on ART (years)	8.5 ± 2.7		
Nadir CD4 count (cells/μl)	113 ± 74		
Baseline CD4 countc (cells/μl)	117 ± 72		
Current CD4 count (cells/μl)	553 ± 251		
Proportion with undetectable VL, n (%)	100		
Time since most recent VL (months)	3.2 ± 2.0		
NNRTI-based ART, n (%)	82 (73)		
PI-based ART, n (%)	29 (26)		

HMG-Co A, 3-hydroxy-3-methylglutaryl-coenzyme A; CVD, cardiovascular disease; LDL, low-density lipoprotein; HDL, high-density lipoprotein; baseline CD4 count, pre-ART CD4 count; VL, viral load; NNRTI, non-nucleoside reverse transcriptase inhibitor; PI, protease inhibitor ^a^Means (standard deviations) for continuous measures; counts (percentages) for categorical measures.

^b^Continuous variables were compared using the two-samples t-test and categorical variables were compared using Fischer’s exact test.

^c^Baseline CD4 count indicates pre-ART CD4 count.

In adjusted analysis, controlling for demographic factors and traditional CVD risk factors, HIV infection was associated with increased log VCAM–1 and ICAM–1 but not with E–selectin levels (p = 0.01, p = 0.03 and p = 0.75, respectively), as shown in Table 2. More traditional risk factors were more significantly associated with VCAM–1 than with ICAM–1 or E–selectin (significant p–values in bold in Table 2).

**Table 2:** Association between VCAM-1, ICAM-1, E-selectin levels and HIV-status in multivariate models (among HIV-infected participants only)

	*Log VCAM-1 (ng/ml)*		*ICAM-1 (ng/ml)*		*^#^E-selectin (ng/ml)*	
*CVD risk factor*	*Estimate (95% CI)*	*p-value*	*Estimate (95% CI)*	*p-value*	*Estimate (95% CI)*	*p-value*
HIV status (ref = controls)	0.14 (0.04, 0.25)	**0.01**	55.17 (5.87, 104.46)	**0.03**	1.19 (–6.15, 8.52)	0.75
Age (per 5-year increase)	0.08 (0.03, 0.13)	< **0.01**	9.03 (–14.62, 32.68)	0.45	–1.59 (–5.07, 1.89)	0.37
Gender (ref = female)	–0.18 (–0.30, –0.06)	< **0.01**	–18.62 (–72.85, 35.61)	0.50	2.49 (–5.72, 10.70)	0.55
Body mass index^a^ (kg/m^2^)	–0.02 (–0.03, –0.01)	< **0.01**	1.81 (–3.52, 7.14)	0.50	0.22 (–0.58, 1.01)	0.59
Mean arterial pressure^b^	0.002 (–0.002, 0.006)	0.44	–0.08 (–1.99, 1.83)	0.93	0.20 (–0.09, 0.49)	0.17
Cholesterol (mmol/dl)	–0.001 (–0.003, 0)	**0.01**	–0.15 (–0.68, 0.38)	0.58	0.01 (–0.08, 0.10)	0.82
Glycosylated haemoglobin (%)	0.01 (–0.06, 0.09)	0.75	20.30 (–14.29, 54.88)	0.25	6.16 (1.43, 10.10)	**0.01**
Previous smoker (not current) vs never	–0.001 (–0.123, 0.121)	0.99	25.02 (–33.28, 83.33)	0.40	4.31 (–4.2, 12.93)	0.33
Current smoker vs never	0.07 (–0.10, 0.23)	0.45	123.93 (44.47, 203.40)	< **0.01**	10.72 (–0.47, 21.90)	0.06
Statin use (ref = on statin)	–0.21 (–0.47, 0.05)	0.12	5.25 (–119.28, 129.78)	0.93	–5.85 (–26.56, 14.85)	0.58

VCAM-1, vascular cell adhesion molecule; ICAM-1, intercellular adhesion molecule.

^#^Significant interaction not reported because of high rates of missing data.

^a^Body mass index calculated as body weight (kg) divided by height^2^ (m).

^b^ Mean arterial pressure calculated as one-third systolic blood pressure (mmHg) plus two-thirds diastolic pressure (mmHg).

In unadjusted analysis, higher log sCD163 was associated with decreased ICAM–1 and E–selectin levels (p < 0.01 and p = 0.01) but not log VCAM–1 (p = 0.07), as shown in Table 3. These associations were linear based on graphical plot. Inclusion of a quadratic term for log sCD163 in each of the ICAM–1, VCAM– 1 and E–selectin models did not alter the reported association (data not shown). In preliminary diagnostics prior to performing adjusted analysis, there was a statistically significant negative correlation between sCD163 and IL–6 (r = –0.27, p < 0.01); however, the estimates were similar between the univariate and adjusted models so both were retained in the final models.

**Table 3:** Association between sCD163, IL-6 levels, cIMT and biomarkers of endothelial dysfunction among HIV-infected participants models

	*Univariate models*		*Multivariable models*	
	*Estimate*	*p-value*	*Estimate (95% CI)*	*p-value*
Log VCAM-1*			Adj R2 = 0.02	
IL-6	–0.0008	0.14	–0.0006 (–0.002, 0.0005)	0.27
Log sCD163*	0.08	0.07	0.08 (–0.02, 0.18)	0.13
cIMT	–0.23	0.54	–0.33 (–1.07, 0.40)	0.37
ICAM-1			Adj R2 = 0.05	
IL-6	0.19	0.40	0.02 (–0.43, 0.47)	0.93
Log sCD163*	–61.7	< 0.01	–61.5 (–105.4, –17.6)	< 0.01
cIMT	–45.1	0.78	1.27 (–309.8, 312.4)	0.99
E-selectin			Adj R2 = 0.05	
IL-6	0.23	0.66	0.15 (–0.86, 1.15)	0.77
Log sCD163*	–7.8	0.01	–8.56 (–15.30, –1.82)	0.01
cIMT	–2.5	0.93	5.64 (–51.2, 62.5)	0.84

VCAM-1, vascular cell adhesion molecule; ICAM-1, intercellular adhesion molecule

*Data log transformed for univariate linear regression analysis for VCAM-1 and sCD163.

Final multivariate models for each marker of endothelial dysfunction demonstrated sCD163 to be associated with decreased ICAM–1 (–61.5, 95% CI: –105.4, 17.6, p < 0.01), and E–selectin levels (–8.56, 95% CI: –15.30, –1.82, p = 0.01) but not log VCAM–1 (0.08, 95% CI: –0.02, 0.18, p = 0.13), as shown in Table 3. These associations were linear. Neither IL–6 nor cIMT was associated with any biomarker of endothelial function.

## Discussion

We found that HIV–infected adults in SSA who had attained viral suppression on ART had biomarkers consistent with greater endothelial dysfunction when compared to HIV–uninfected controls, even after adjusting for traditional CVD risk factors. In this small cross–sectional study of adults between 30 and 50 years, endothelial dysfunction was associated with sCD163 but not IL–6 or cIMT.

This evidence of ongoing endothelial dysfunction despite chronic viral suppression among HIV–infected patients when compared to HIV–uninfected controls indicates that endothelial dysfunction, first observed prior to ART initiation in this setting,^2^ persists following ART initiation, similar to what has been observed in other settings.^5^–^7^ Specifically, persistently elevated ICAM–1 levels following viral suppression were observed among black South Africans,^28^ Kenyan women^29^ and a predominantly male Danish cohort^7^ following mean ART duration of approximately three, one and 12 years, respectively. This aligns well with our findings following a mean of nine years of ART exposure.

Furthermore, our finding of elevated VCAM–1 levels following prolonged ART was previously observed in the same black South African cohort studied by Fourie et al.,^28^ but not in the Danish cohort.^7^ To the best of our knowledge, our findings support those of Fourie et al. in a mixed male and female cohort and expand on these findings by providing ascertainment of date of ART initiation.^28^ Taken together, our findings in relation to ICAM–1 and VCAM–1 build on those of Fourie et al.^28^ and Graham et al.^1^ in SSA and also highlight the strong effect of HIV disease on endothelial dysfunction, even after prolonged viral suppression and controlling for multiple traditional CVD risk factors and the use of statin therapy.

We did not observe an association between HIV status and elevated E–selectin level, in keeping with a similar report in a Danish cohort of virally suppressed HIV–patients versus controls.^7^ In the Graham report among Kenyan women, E–selectin levels were unchanged at 12 months compared to baseline.^1^ We believe our report of a lack of difference in E–selectin levels by HIV status to be the first in SSA. The Graham and Danish cohort reports suggest that HIV may not be as strongly associated with E–selectin, compared to VCAM–1 or ICAM–1 levels.

Our findings of a lack of association between IL–6 level and cIMT with endothelial dysfunction are similar to those of Fourie et al. in their study among virally suppressed black South African patients.^28^ Our findings and those of Fourie et al. in this context do not align with the observation among non–African patients that IL–6 level and cIMT were associated with endothelial injury.^8^,^26^,^30^ Our small sample size may have precluded our ability to detect these associations in our setting.

Of note, observations of this association in non–African cohorts have not always been consistent. In a recent prospective study of ART–naive HIV–infected adults in the USA, ICAM–1 level was not associated with progression in cIMT.^31^ More studies are required to establish the contribution of traditional CVD risk factors to endothelial dysfunction among virally suppressed HIV–infected patients in SSA, and to explore novel markers that may explain the proportion of risk not conferred by traditional CVD risk factors.

The relationship between sCD163 level and biomarkers of endothelial dysfunction has not been reported in SSA. Our results of a lack of association between sCD163 and VCAM–1 have been reported by some,^32^ but not all^33^ studies in non–SSA settings among patients on ART. We had expected a positive association between sCD163 and VCAM–1 in our study because sCD163 induces arterial inflammation, which may lead to increased shedding of VCAM–1.^26^ We observed an unexpected negative association between sCD163 with I–CAM and E–selectin levels. We are cautious in interpreting the negative associations observed between sCD163 with ICAM–1 and E–selectin levels in our study pending assessment of these associations in similar cohorts.

## Limitations

The main limitations of our study are the cross–sectional design, small sample size and limited number of biomarkers that were studied. The cross–sectional design precludes the ability to arrive at a temporal association between our predictors and outcomes. Furthermore, studying activated peripheral blood mononuclear cells and human endothelial cells would provide a more robust understanding of pathways involved in the development of endothelial dysfunction. Because we relied on standard–ofcare HIV RNA results that were measured within an average of three months prior to time of enrolment, it is possible that some patients may have been viraemic. We believe that this potential effect would have been minimal, given the high viral suppression rates of 96.5% observed among patients on ART in routine HIV care in Botswana.34 A strength of our study was the ascertainment of traditional CVD risk factors, which allowed for robust assessment of confounding.

## Conclusion

We observed that HIV disease was associated with biomarkers of endothelial dysfunction among virally suppressed adults in Botswana on long–term ART after controlling for traditional CVD risk factors. Unexpectedly, we observed a modest inverse relationship between sCD163 level (a marker of monocyte activation) and some biomarkers of endothelial dysfunction. Our study is the first to report on markers of endothelial dysfunction following prolonged ART among virally suppressed black Africans. We also report a lack of association between sCD163 and VCAM–1 for the first time in a SSA clinical cohort, replicating what has been observed in non–African cohorts, and we corroborate a previously demonstrated lack of association between IL–6 and cIMT with endothelial dysfunction in this setting. Future work should explore the impact of persistent endothelial dysfunction following long–term HIV viral suppression on the risk of CVD clinical endpoints among HIV–infected patients in this setting.
